# Thirty-Sixth Annual Commencement of the Baltimore College of Dental Surgery

**Published:** 1876-04

**Authors:** 


### ARTICLE VI.

*The Thirty Sixth Annual Commencement of the Baltimore College of Dental Surgery*, was held at the Academy of Music, Baltimore City, on Friday evening, March 10th, 1876, in the presence of a large and brilliant audience. The stage was occupied by the Faculty, Graduating Class, a number of the Professors of the several medical colleges of the city, and invited guests. Prof. Wernig's Fifth Regiment Orchestra furnished a number of select pieces of music.

The exercises commenced with prayer, by Rev. Dr. J. B. Purcell. An announcement of the present condition of the College, followed by the names of the graduates, was made by the Dean, after which the degree of "Doctor of Dental Surgery" was conferred upon the following students by Prof. F. J S. Gorgas :

SUBJECT OF THESIS.

Rufus Calvin Bowman, Virginia.—Treatment Preparatory to Filling Teeth.
George Crowther, *South America.*—Mechanical Dentistry.
Herbert E. Dennett, *Massachusetts.*—Lining the Walls.
John P. Dennett, *Massachusetts.*—Mechanical Dentistry.
Edward P. Doremus, *Louisiana.*—Dental Caries and its Causes.
William Henry Dwinelle, *New York.*—Gold.—Its Relation to Dentistry.
Richard Atwell Fox, *Virginia.*—Development and Structure of the Teeth.
James Allen Glenn, *North Carolina.*—Circulation of the Blood.
Elise von Heyden, *Prussia.*—Secondary Dentine.
Samuel Kimmell, *Maryland.*—Nitrous Oxide Gas.

William Samuel Krebs, *Maryland.*—Anæsthetics and their Application to Dentistry.
Ezekiel Daniel Margary, *West Indies.*—The Extraction of Teeth.
Brainerd T. Olcott, *New Hampshire.*—The Effect of the Diet upon the Teeth.
William La Fayette Seigler, *Florida.*—The Teeth.
Wharton Hume Shine, *Florida.*—Digestion.
Augustus Wilson Sweeny, Jr., *Maryland.*—The Relation of Dentistry to Medicine.
William Oscar Thrailkill, *Kentucky.*—Injuries to the Teeth and Soft Structures by Mechanical Violence.
Otho Frank Welsh *Virginia.*—Mechanical Dentistry.
Gurdon F. S. Wright, *South Carolina.*—The Bicuspids.

The Valedictory Address was then delivered by Prof. E. Lloyd Howard, M. D., followed by a Class Address by James Allen Glenn, of North Carolina.

The exercises closed with the benediction by Rev. Dr. Purcell. The number of matriculants during the session of 1875–76, was *fifty-two.* The Spring session of 1876, will commence on the 1st of April and continue until the last of June following. The Winter session will commence on the 17th of October, 1876, and continue until March 1877.

---

